# Swimming Back and Forth Using Planar Flagellar Propulsion at Low Reynolds Numbers

**DOI:** 10.1002/advs.201700461

**Published:** 2017-12-01

**Authors:** Islam S. M. Khalil, Ahmet Fatih Tabak, Youssef Hamed, Mohamed E. Mitwally, Mohamed Tawakol, Anke Klingner, Metin Sitti

**Affiliations:** ^1^ Department of Mechatronics Department of Materials Engineering Department of Physics German University in Cairo New Cairo 11835 Egypt; ^2^ Physical Intelligence Department Max Planck Institute for Intelligent Systems Stuttgart 70569 Germany

**Keywords:** biologically inspired microrobots, flagellar propulsion, magnetic microparticles, soft microrobots

## Abstract

Peritrichously flagellated *Escherichia coli* swim back and forth by wrapping their flagella together in a helical bundle. However, other monotrichous bacteria cannot swim back and forth with a single flagellum and planar wave propagation. Quantifying this observation, a magnetically driven soft two‐tailed microrobot capable of reversing its swimming direction without making a U‐turn trajectory or actively modifying the direction of wave propagation is designed and developed. The microrobot contains magnetic microparticles within the polymer matrix of its head and consists of two collinear, unequal, and opposite ultrathin tails. It is driven and steered using a uniform magnetic field along the direction of motion with a sinusoidally varying orthogonal component. Distinct reversal frequencies that enable selective and independent excitation of the first or the second tail of the microrobot based on their tail length ratio are found. While the first tail provides a propulsive force below one of the reversal frequencies, the second is almost passive, and the net propulsive force achieves flagellated motion along one direction. On the other hand, the second tail achieves flagellated propulsion along the opposite direction above the reversal frequency.

## 1 Introduction

In recent years, considerable progress has been achieved in the development of biologically inspired robots at the micro‐ and nanoscales. Several research groups have utilized diverse micro‐ and nanofabrication techniques to complement basic analytical studies^1^, ^2^, ^3^, ^4^ on the motility of microorganisms by experimental work.^5^, ^6^, ^7^, ^8^, ^9^, ^10^ The motility of monotrichous flagellated microorganisms has been mimicked, and controlled locomotion using helical^11^ and planar^12^, ^13^, ^14^, ^15^ travelling waves have been experimentally demonstrated. Very recently, motility of peritrichously flagellated microorganisms has also been mimicked using a magnetically actuated multilink nanoswimmer.^16^ Huang et al.^17^, ^18^ have also integrated self‐folded hydrogel tubes to artificially approximated bacterial flagella to enhance the overall motility of the soft microrobot. Lum et al. have proposed a universal programming methodology to achieve desired magnetization profiles for soft materials and enable realization of time‐varying shapes.^19^ Palagi et al. have demonstrated plane wave propagation and swimming of photoactive soft robots actuated by different patterns of incident light.^20^ Xu et al. have also developed millimeter‐scaled swimmers with superhydrophobic and superhydrophilic soft tails to investigate the influence of the surface properties on the swimming characteristics.^21^ While the mechanics of the flagellum has been replicated by an elastic tail, this design has restricted the motion of one‐tailed microrobots to be along one‐direction. The one‐tailed microrobot has to undergo a U‐turn trajectory with relatively large curvature (a few body lengths) to move along the opposite direction. Unlike rotation of a helical filament that can result in back and forth locomotion based on the direction of rotation, the actuation of a flexible tail to generate propulsive force does not enable back and forth locomotion without a U‐turn trajectory with relatively large curvature. This behavior represents a physical limit on the maneuverability of one‐tailed microrobots. The implication is that any one‐tailed microrobot must undergo a U‐turn trajectory to move in the opposite direction. In several biomedical applications, it is likely that these microrobots will swim along narrow channels or capillaries that restrict a U‐turn trajectory, and hence limit the usefulness of these microrobots in nontrivial tasks that necessitates motion reversals.

**Figure 1:**
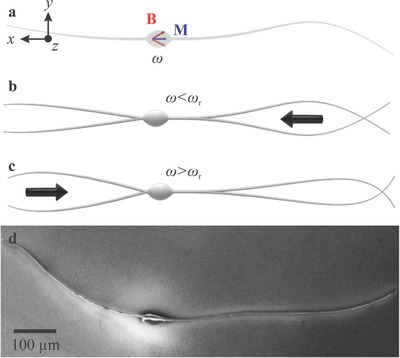
A soft two‐tailed microrobot with a magnetic head and two collinear, unequal, and opposite tails is fabricated using electrospinning. a) Magnetic particles are incorporated within the head to provide magnetization (M), and directional control under the influence of a periodic magnetic field (B) at frequency ω. b) Below reversal frequency (ω_r_), the net propulsive force enables the microrobot to swim using its long tail. c) Above ω_r_, the short tail allows for swimming along the opposite direction. d) A scanning electron microscopy image shows the morphology of the two‐tailed microrobot.

Here we are instead interested in achieving back and forth flagellated propulsion without a U‐turn trajectory. We propose a simple design of a soft two‐tailed microrobot that consists of a magnetic head and two collinear, unequal, and opposite ultrathin tails (**Figure** **1**a). In contrast to flagellated propulsion using single flexible tail, the incorporation of a second tail provides frequency dependent propulsive force that enables selective reversal of the swimming direction based on the applied frequency and the length ratio of the tails (Figure 1b,c).

## 2 Swimming Back‐and‐Forth Using Planar Wave Propagation

### 2.1 Theoretical Model

The incorporation of nonidentical flexible tails to a magnetic head results in two opposite propulsive forces. The elasticity of each tail determines the direction of propulsion based on the frequency of oscillation. The numerical model of the mentioned microrobotic configuration includes three components: elastohydrodynamics for dynamic deformations, magnetohydrodynamics based on the Biot–Savart law for the magnetic actuation of the head,^22^ and rigid‐body kinematics based on steady‐periodic Stokes‐flow approach with force free swimming conditions. Each component utilizes resistive‐force coefficients based on the resistive‐force theory (RFT) to calculate the resultant hydrodynamic forces acting on the elastic tails and the magnetic head of the microrobot.^23^ The structural deformation in the frame of the two‐tailed microrobot with reference (ϕ_*i*_) is modeled based on the Timoshenko–Rayleigh beam theory to predict relatively high‐frequency effects of the moment of inertia and axial shear within the elastic tails^24^, ^25^

(1) $$E_{i}\;I_{i}\;\frac{\partial^{4}\varphi_{i}}{\partial x^{4}}\;\;=\;\;F_{iy}$$

where *E*_*i*_ is the Young's modulus of the *i*th tail, for *i* = 1, 2. Further, *F*_*iy*_ denotes the lateral local forces acting on the *i*th elastic tail due to fluid drag, cross‐sectional torsion, and inertial effects. *F*_*iy*_ provides the necessary elasto‐hydrodynamic effects pertaining to the structural deformation and resultant fluid resistance. The Rayleigh–Timoshenko beam model is used to account for inertial and shear effects as plane‐wave propagation occurs (see the Experimental Section). We consider that the tails are flexible but not extensible, hence their lengths ($l_{t_{i}}$) remain constant. However, the *x*‐coordinates along the tail are subject to deformation. The equation of motion of the two‐tailed microrobot based on the force‐free swimming condition is given by

(2) $$\left(\begin{array}{c} V\\ \Omega \end{array}\right)\;\;=\;\;-Z_{r}^{-1}\left(\begin{array}{c} V\left(M\cdot \nabla \right)(R_{r}B)\\ V\;M\;\;\times \;\;(R_{r}B) \end{array}\right)$$

where V and Ω are the linear and angular rigid‐body velocities of the microrobot, respectively. $Z_{r}$ is the overall hydrodynamic resistance matrix of the microrobot (see the Experimental Section). The microrobot is actuated using a periodic magnetic field B to exert torque on its magnetic dipole (M), and the rotation matrix $R_{r}$ projects the magnetic field onto the frame of reference of the microrobot. *V* is the volume of the magnetic particles within the polymer matrix of its head. Flagellar propulsion of the microrobot is mainly due to the external magnetic torque generated using in‐plane magnetic field. To understand the influence of having two tails, we consider the propagation of a wave *A*_0_ cos (*ωt* − *kx*), where *k* is the wave number and *A*_0_ is the maximum deformation. The microrobot swims along its long axis. Therefore, the components of the net propulsive force $f_{x_{1}}\;\;-\;\;f_{x_{2}}\;\;=\;\;\frac{\omega }{2\pi }\int_{2\pi /\omega }f_{x_{1}}(t)\;\;-\;\;f_{x_{2}}(t)dt$, along direction of motion are given by

(3) $$f_{x_{i}}=\int_{0}^{l_{t_{i}}}\frac{-v_{x}\left(C_{t_{i}}+\beta_{i}^{2}C_{n_{i}}\sin{\left(k_{i}x-\omega t\right)}^{2}\right)-\beta_{i}v_{y}\sin\left(k_{i}x-\omega t\right)(C_{n_{i}}-C_{t_{i}})}{\beta_{i}^{2}\sin{\left(k_{i}x-\omega t\right)}^{2}+1}dx$$

where $f_{x_{1}}$ and $f_{x_{2}}$ are the propulsive forces of the first and second tails, respectively. *v*_*x*_ and *v*_*y*_ are the forward and lateral speeds of the microrobot, respectively. Further, $C_{t_{i}}\;\;=\;\;-2\pi \;\mu /(\ln\left(\frac{l_{t_{i}}}{r_{t_{i}}}\right)\;\;-\;\;0.807)$ is the tangential drag coefficient of *i*th tail, and $C_{n_{i}}\;\;=\;\;-4\pi \;\mu /(\ln\left(\frac{l_{t_{i}}}{r_{t_{i}}}\right)\;\;+\;\;0.193)$ is its normal drag coefficient. The constants μ and $r_{t_{i}}$ are the viscosity of the medium and the radius of the *i*th tail, respectively, and $\beta_{i}\;\;=\;\;k_{i}\;A_{0_{i}}$. Equation (3) represents the theoretical limit of pure sinusoidal wave propagation as opposed to a decaying wave amplitude from the head to the tip of the elastic tail. If we assume that the two tails are identical, i.e., $l_{t_{1}}\;=\;\;l_{t_{2}}$ and $r_{t_{1}}\;=\;\;r_{t_{2}}$, then the microrobot will have zero net propulsive force regardless to the actuation frequency ω. On the other hand, the sum of the propulsive forces ($f_{x_{1}}\;-\;\;f_{x_{2}}$) will be positive or negative when the tails are not identical, for a fixed actuation frequency. Another important consequence of the deviation in length between the tails for varying actuation frequency is that the sum of propulsive forces will be zero at certain frequencies, which we refer to as the reversal frequencies ω_r_. These reversal frequencies are dependent on the length ratio of the tails, and represent the roots of $f_{x_{1}}\;\;-\;\;f_{x_{2}}\;\;=\;\;0$, for nonidentical tails, i.e., $l_{t_{1}}\neq \;\;l_{t_{2}}$. For example, below ω_r_ the microrobot will achieve planar flagellar propulsion along one direction, while above ω_r_ it will reverse its direction.

### 2.2 Determination of Microrobot Parameters

**Figure 2:**
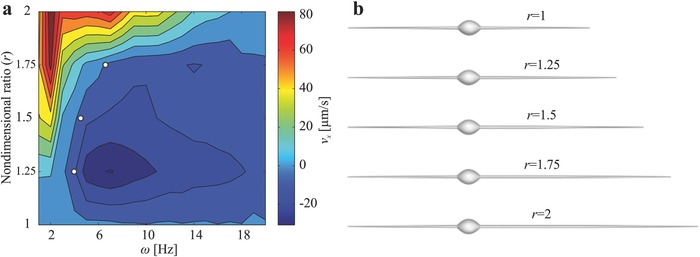
Prediction of the swimming direction of the soft two‐tailed microrobot by the model represented by Equations (1) and (2). a) The map of the predicted forward velocity (*v*_*x*_) of the microrobot indicates distinct frequencies (ω) to achieve back and forth motion using planar flagellar propulsion, for a microrobot with a 215 µm long first tail, and 25 and 80 µm in minor and major head diameter, respectively. The second tail length ranges between 215 and 430 µm. The three white dots indicate that microrobots with length ratio (*r*) of 1.25, 1.5, and 1.75 change the direction of motion at frequencies of 3.2, 4.2, and 6.4 Hz, respectively. b) Five groups of microrobots are fabricated based on the simulation results with tail length ratios of 1, 1.25, 1.5, 1.75, and 2.

We solve Equations (1) and (2) to determine microrobot parameters that enable back and forth flagellated swim, and calculate the average velocity for a range of actuation frequencies and different length ratio between the first and second tails. The dimensions and magnetization of the magnetic head and the diameter and modulus of elasticity of the tails are fixed in this calculation. Stiffness of each tail is influenced mainly by its length. A distinct parametric space of actuation frequencies is predicted, as shown in **Figure** **2**a. The theoretical model shows that two‐tailed microrobot with tails of equal lengths (*r* = 1) has a negligible velocity (*v*_*x*_) along the direction of propulsion regardless to the actuation frequency. As we increase the length of one tail and keep the other fixed, not only do we observe that the velocity of the microrobot changes with the frequency, but we also observe that the frequency dictates the direction of propulsion. At *r* = 1.25, the microrobot swims along one direction using its long tail below actuation frequency of 3.2 Hz. Above this reversal frequency (ω_r_), the short tail is responsible for propulsion along the opposite direction. We also find that the reversal frequency is shifted to 4.2 Hz when one tail is 50% longer than the other. At this ratio (*r* = 1.5), the long tail provides greater propulsive force than the short tail for ω < ω_r_, then the short tail achieves motion reversal above ω_r_. For ratio *r* = 1.75, our model predicts a reversal frequency at 6.4 Hz. Finally, the microrobot does not reverse its direction when one of its tails is twice the length of the other (*r* = 2). At this ratio, the long tail is responsible for propulsion throughout the actuation frequency range.

### 2.3 Experimental Results

**Figure 3:**
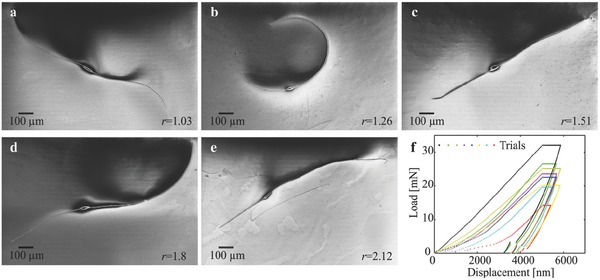
Five groups of two‐tailed microrobots are fabricated with tail length ratio *r*. a) *r* = 1.08 ± 0.08 (*n* = 6). b) *r* = 1.24 ± 0.11 (*n* = 6). c) *r* = 1.48 ± 0.08 (*n* = 6). d) *r* = 1.71 ± 0.09 (*n* = 6). e) *r* = 2.14 ± 0.16 (*n* = 6). Conditions: polymer concentration 25 wt% in DMF, weight ratio iron:polystyrene 1:2, electric field 25 kV, and flow rate 20 µL min^−1^ (see the Experimental Section). f) Load–displacement curves of the nanoindentation experiment are used to characterize the modulus of elasticity (see the Experimental Section). The modulus of elasticity of the microrobot is 0.58 ± 0.054 GPa (*n* = 8).

Two‐tailed microrobots are fabricated based on the theoretical model and the simulation results (Figure 2b). The microrobots are prepared by electrospinning a solution of polystyrene in dimethylformamide (DMF) and magnetic particles with polymer concentration of 25 wt% in DMF, weight ratio iron:polystyrene 1:2, electric field 25 kV, and flow rate 20 µL min^−1^. Five groups of microrobots are fabricated with tail length ratio $\left(r=\frac{l_{t_{1}}}{l_{t_{2}}}\right)$ of ≈1, 1.25, 1.5, 1.75, and 2, as shown in **Figure** **3**a–e. The modulus of elasticity of the microrobots is characterized by depth‐sensing indentation, as shown in Figure 3f. The modulus of elasticity is determined using the load‐displacement curves to be 0.58 ± 0.054 GPa (*n* = 8). Each group of microrobots is contained inside a deep chamber that contains glycerine with viscosity of 0.95 Pa s. For these microrobots, Reynolds number is on the order of 10^−5^ and is calculated as $Re=\frac{\rho v_{x}L}{\mu }$, where ρ is the density of the medium and *L* is the total length. The microrobots are observed using a microscopic unit (MF Series 176 Measuring Microscopes, Mitutoyo, Kawasaki, Japan). Videos are acquired using a camera (avA1000‐120kc, Basler Area Scan Camera, Basler AG, Ahrensburg, Germany) and a 3× Mitutoyo phase objective. Actuation of the microrobot is achieved using in‐plane magnetic fields. These fields are generated using four orthogonal electromagnetic coils to provide uniform field along direction of motion of the microrobot and with a sinusoidally varying orthogonal component to achieve oscillation (see the Experimental Section). The electromagnetic configuration provides maximum magnetic field gradient of 5 T m^−1^ and magnetic field with almost similar magnitude (18 mT) within a frequency range of 1–20 Hz. Therefore, we limit the frequency of the actuating field to this range. The system also generates a magnetic field gradient that does not influence the propulsion of the microrobots. The difference between the drag and magnetic forces along direction of propulsion is on the order of O(10^−4^) µN. Therefore, propulsion of the microrobot is solely due to its beating flexible tails.

**Figure 4:**
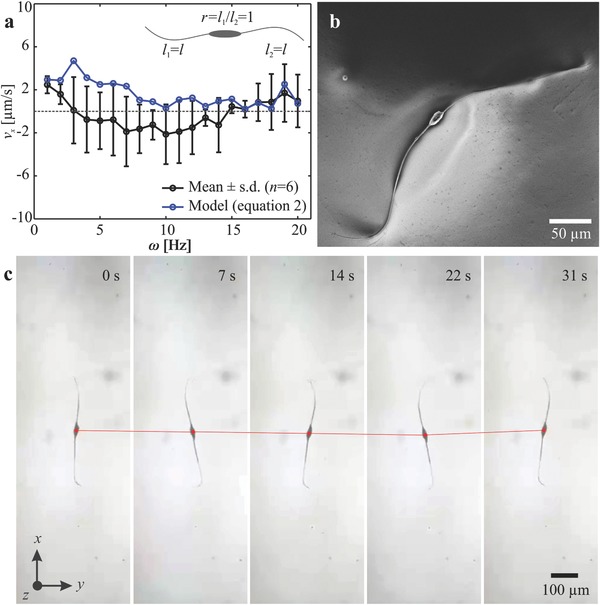
Frequency response of two‐tailed microrobots with tail length ratio *r* = 1. a) The microrobots exhibit negligible motion as the two propulsive forces of the almost equal tails (*l*_1_ ≃ *l*_2_ ≃ *l*) are similar and act along opposite directions. b) A scanning electron microscopy image of a microrobot with two tails with almost equal length. c) Sequence of microrobot displacement at frequency 1 Hz shows that the speed is 1.3 µm s^−1^ (Movie S1, Supporting Information).

**Figure** **4** shows the response of two‐tailed microrobots with tail length ratio *r* ≃ 1. The propulsive forces generated by the equal tails do not enable the microrobot to swim along its long axis throughout the actuation frequency range, as shown in Figure 4a. Figure 4b provides a representative microrobot from this group with a tail length ratio of 1.06. The deviation in length between its tails results in a nonzero velocity of the microrobots. The deviations in radius between the first (4.1 µm) and second tails (5.3 µm) are entered to the theoretical model and result in a nonzero velocity. We also observe a deviation in the swimming direction between our experiments and theoretical prediction within actuation frequency range of 4–14 Hz. This deviation is attributed to the geometric aberrations along the tails and can be decreased by accurate measurement of the resistive‐force coefficients of each microrobot. Nevertheless, microrobots with tail length ratio of 1 achieve negligible net displacement along the direction of motion. At actuation frequency of 1 Hz, the microrobot swims at an average speed of 1.3 µm s^−1^, which is equivalent to 2.6 × 10^−3^ of its body length per second. Therefore, the incorporation of almost identical tails at both ends of the head cancels out the propulsive forces and results in a negligible net displacement of the microrobot, as shown in Figure 4c (Movie S1, Supporting Information).

**Figure 5:**
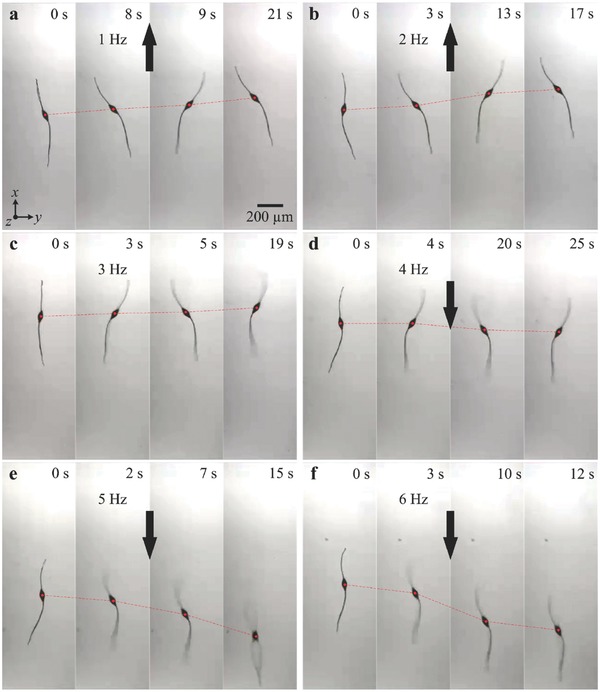
A two‐tailed microrobot with tail length ratio *r* ≃ 1.5 achieves planar flagellar propulsion along opposite directions using two actuating frequency ranges. a,b) At frequencies of 1 and 2 Hz, the long tail provides greater propulsive force and the microrobot swims at average speed of 8.1 and 19.1 µm s^−1^, respectively. c) Although the tails are not equal in length, they provide approximately equal propulsive force at 3 Hz and the speed of the microrobot is decreased to 3.9 µms^−1^. d–f) At frequencies of 4, 5, and 6 Hz, the short tail provides greater propulsive force and the microrobot reverses its direction and swims at average speed of −0.45, −9.7, and −35.2 µm s^−1^, respectively (Movie S2, Supporting Information).

**Figure 6:**
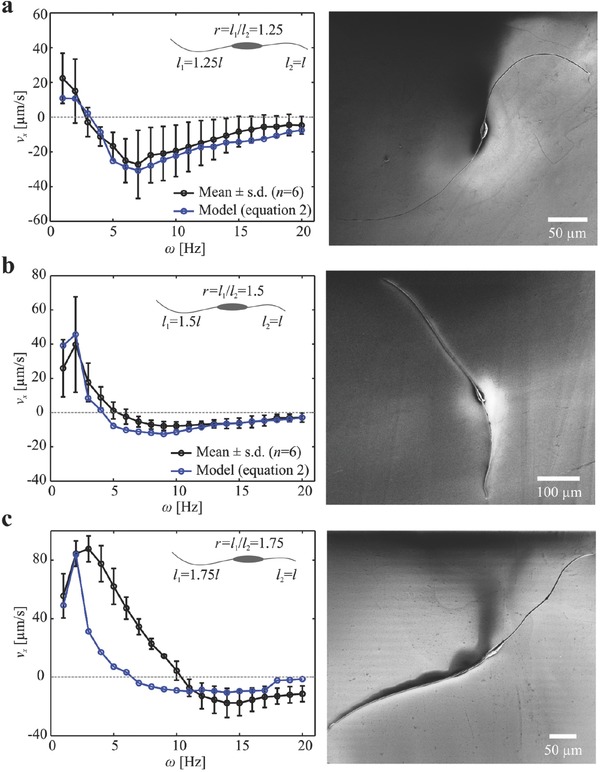
Frequency response of microrobots with different tail length ratio (*r* ≃ 1.25, *r* ≃ 1.5, and *r* ≃ 1.75). The reversal frequency varies with the tail length ratio of each microrobot. a) Microrobots with tail length ratio 1.25 ± 0.05 (*n* = 6) have reversal frequency of 2.8 ± 0.9 Hz (*n* = 6). b) Microrobots with tail length ratio 1.5 ± 0.08 (*n* = 6) have reversal frequency of 5.4 ± 0.7 Hz (*n* = 6). c) Microrobots with tail length ratio 1.75 ± 0.03 (*n* = 6) have reversal frequency of 10.4 ± 0.5 Hz (*n* = 6) (Movie S2, Supporting Information).

**Figure 7:**
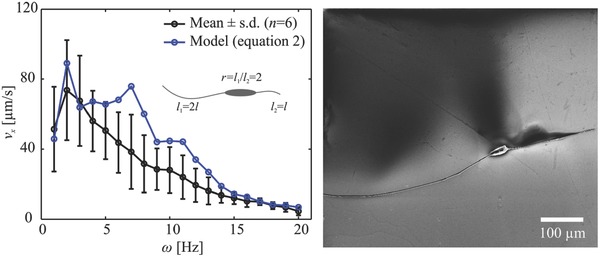
Frequency response of two‐tailed microrobots with tail length ratio (*r* ≃ 2). The frequency of the external magnetic field does not influence the swimming direction as the longer tail provides greater propulsive force throughout the whole frequency range (Movie S3, Supporting Information).

Now we turn our attention to two‐tailed microrobots with nonidentical tails. **Figure** **5** shows the response of a microrobot with ratio of 1.5 to a periodic magnetic field. The length of the short and long tails are 360 and 242 µm, whereas the minor and major diameters of the head are 39 and 95 µm, respectively. We observe that the propulsive force of the long tail is greater than that of the short tail at low actuation frequencies (1 and 2 Hz), as shown in Figure 5a,b. At ≈4 Hz, the two tails generate equal propulsive force and negligible displacement along the long axis of the microrobot is observed (Figure 5c). Therefore, the reversal frequency of this microrobot is ≈3 Hz. The short tail becomes dominant at relatively high actuation frequencies (4–6 Hz). The average speed of the microrobot is calculated to be −0.45, −9.7, and −35.2 µm s^−1^ for actuation frequency of 4, 5, and 6 Hz, respectively, as shown in Figure 5d–f (Movie S2, Supporting Information). Frequency response of microrobots with nonidentical tail length is summarized in **Figure** **6**. The measured reversal frequency (ω_r_) increases with the tail length ratio. A reversal frequency of 2.8 ± 0.9 Hz (*n* = 6) is measured for microrobots with tail length ratio *r* = 1.24 ± 0.11 (*n* = 6), and increases to 5.4 ± 0.7 Hz (*n* = 6) and 10.4 ± 0.5 Hz (*n* = 6) for tail length ratios of 1.48 ± 0.08 (*n* = 6) and 1.71 ± 0.09 (*n* = 6), respectively. This response indicates that as one tail decreases in length, the reversal frequency increases and enables the long tail to have wider range to control the speed of the microrobot along one direction. The frequency response of the microrobots also shows relatively large variation in swimming speed for a slight difference in the actuation frequency below ω_r_, whereas the influence of the actuation frequency becomes negligible above ω_r_, as shown in Figure 6. By increasing the tail length ratio to *r* ≃ 2, we observe that the long tail provides greater propulsive force throughout the actuation frequency range, as shown in **Figure** **7** (Movie S3, Supporting Information).

## 3 Discussion

In this study we show the feasibility of two‐tailed microrobots to swim back and forth using planar flagellar propulsion. The configuration of the microrobot which includes collinear nonidentical flexible tails enables propulsion along opposite directions using distinct reversal frequencies. These frequencies depend on the ratio between the tail lengths of the microrobot and provide two frequency ranges (for each tail length ratio) to allow motion reversals without U‐turn trajectory. Our hydrodynamic model provides qualitative agreement with our experimental results with notable quantitative agreement for ratios *r* = 1.25 and *r* = 1.5. All parameters entered to this model are determined independently of the measured wave variables of the flexible tails, swimming speed, and reversal frequencies. Geometric parameters are determined using scanning electron microscopy images and the average lengths of each tail, average minor and major head diameters, average tail radius, and average modulus of elasticity are entered to the model. Therefore, we attribute the deviations between the theoretical predictions and experimental results to error in these measurements. These errors influence our theoretical predictions as RFT for wave propagation is based on time‐averaged analysis that necessitates accurate measurement of the parameters.

Although the incorporation of two nonidentical tails enables the microrobots to swim back and forth, we do not observe a notable decrease in their average speed compared to one‐tailed microrobots. Our experimental results on one‐tailed microrobots show that planar flagellar propulsion achieves 0.05 and 0.06 body length per second under the influence of actuation frequency of 1 and 2 Hz, respectively, whereas the two‐tailed microrobots with ratio *r* ≃ 1.25 achieve average speed of 0.03 and 0.02 body length per second at ω = 1 Hz and ω = 2 Hz, respectively. The difference in average speeds between the one‐ and two‐tailed microrobots becomes smaller for ratio *r* ≃ 1.5. At this ratio, the two‐tailed microrobot swims at 0.04 and 0.06 body length per second at ω = 1 Hz and ω = 2 Hz, respectively. For ratios *r* ≃ 1.75, the two‐tailed microrobot swims at 0.072 and 0.11 body length per second at ω = 1 Hz and ω = 2 Hz, respectively. Therefore, at this ratio the two‐tailed microrobots are not only faster than the one‐tailed microrobots but also have the ability of reversing their direction.

Our experimental frequency response results show another interesting difference between the one‐ and two‐tailed microrobots during motion reversal. The one‐tailed microrobot does not exhibit change in swimming speed along its U‐turn trajectory to reverse its direction, whereas the two‐tailed microrobots relies on the actuation frequency in their motion reversal. For ratio *r* = 1.25, two‐tailed microrobot can swim at average speed of 0.01 body length per second along the opposite direction at 7 Hz. This average speed increases to 0.013 and 0.023 body length per second at 10 and 15 Hz for two‐tailed microrobot with ratio of 1.5 and 1.75, respectively. Therefore, two‐tailed microrobot with ratio of 1.5 and 1.75 swims at approximately twice the speed of the one‐tail microrobot but only along one direction using relatively low actuation frequency. However, they swim at approximately half the speed of the one‐tailed microrobot at relatively high actuation frequency. In contrast to ratios 1.5 and 1.75, two‐tailed microrobot with ratio of 1.25 does not exhibit relatively large variation in maximum swimming speed along the opposite direction as it swims at 0.04 body length per second at ω = 7 Hz.

To conclude, we demonstrate the feasibility of swimming back and forth using planar flagellar propulsion. A two‐tailed microrobot is designed, developed, and its capability to reverse its motion selectively using external magnetic field is demonstrated. The combination of two tails overcomes limitations encountered by one‐tailed microrobot during motion reversals. These reversals are necessary when the microrobots are limited by a constraint force during targeted therapy or several biomedical applications in vessels or capillaries. Our study provides the theoretical elements to model, design, fabricate, and control the two‐tailed microrobots. Although polystyrene is used to fabricate the microrobots, electrospinning of beaded fibers using biocompatible materials is applicable. Actuation of the microrobot is achieved using magnetic field in millitesla range. Therefore, the microrobots can be visualized and controlled using magnetic resonance imaging system.^27^

## 4 Experimental Section

*Microrobot Fabrication and Preparation*: The fabrication of the soft two‐tailed microrobots was done by pumping a polymer solution, i.e., polystyrene (168 N, BASF AG) in DMF and magnetic particles with maximum diameter of 30 µm, into a syringe (polymer concentration is 25 wt% in DMF, weight ratio of iron:polystyrene is 1:2). A strong electric potential was applied between the syringe and a collector using field of 25 kV at distance 10 cm, to draw the solution toward the collector. A syringe pump was used to inject the polymer solution at flow rate of 20 µL min^−1^. The applied electric potential and the distance between the syringe needle and the collector yielded electric gradient of 100 kV m^−1^. This process resulted in the development of beaded fibers, and the microrobots shown in Figure 3 were produced.

*Measurement of Parameters*: Depth sensing indentation was used to measure the Young's modulus (*E*) of the soft microrobots. The depth of indentation was limited to 10% of the minor diameter of the head to avoid any interaction with the substrate beneath the microrobots.^28^ A spherical diamond tip with a radius of 25 µm and Young's modulus *E*_*i*_ = 1141 GPa was used to make 8 indentations at different locations within the head of the microrobot with a hold time of 30 s at the maximum load. Young's modulus was calculated via the unloading stiffness using the Oliver–Pharr method from the resulting load–displacement curves (Figure 3f)

(4) $$E\;\;=\;\;\frac{1-v_{s}^{2}}{\frac{1}{E_{r}}-\frac{1-v_{i}^{2}}{E_{i}}}$$

where *v*_s_ = 0.2 is the Poisson's ratio of the sample and *v*_i_ = 0.07 is the Poisson's ratio of the diamond tip. Further, *E*_r_ is the reduced modulus given by

(5) $$E_{r}=\frac{\sqrt{\pi }}{2}\frac{dp}{dh}\frac{1}{\sqrt{A}}$$

where $\frac{dp}{dh}$ is the unloading stiffness calculated from the load–displacement curves, and *A* is the projected contact area between the diamond tip and the sample at the maximum load. Based on Equations (4) and (5), the average Young's modulus is 0.58 ± 0.054 GPa. The calculated Young's modulus was comparable to the Young's modulus of pure electrospun polystyrene fibers which had an average of 0.68 GPa for 1.76 µm diameter fibers.^29^ The slight deviation in our measurement was due to the bigger diameter of the head compared to a 1.76 µm fiber since Young's modulus decreased with diameter.^30^

*Fluidic and Structural Effects*: The forces acting on the elastic tails due to drag, torsion, and inertia are calculated using

(6) $$\begin{array}{l} F_{iy}=f_{y}+\left(J_{i}+\frac{m_{i}E_{i}I_{i}}{k_{i}A_{i}G_{i}}\right)\frac{\partial^{4}\varphi_{i}}{\partial t^{2}\partial x^{2}}+\frac{J_{i}}{k_{i}A_{i}G_{i}}\frac{\partial^{2}f_{y}}{\partial t^{2}}\\ -\frac{E_{i}I_{i}}{k_{i}A_{i}G_{i}}\frac{\partial^{2}f_{y}}{\partial x^{2}}-m_{i}\frac{\partial^{2}\varphi_{i}}{\partial t^{2}}-\frac{J_{i}m_{i}}{k_{i}A_{i}G_{i}}\frac{\partial^{4}\varphi_{i}}{\partial t^{4}} \end{array}$$

where *A*_*i*_, *I*_*i*_, *m*_*i*_, and *J*_*i*_ are the cross‐section area, second moment of area, mass per unit length, and moment of inertia per unit length of the *i*th tail, respectively, and *k* is the shape correction coefficient for Timoshenko's beam theory. Further, *G*_*i*_ is the shear modulus of the *i*th tail. For each tail, the following boundary condition is imposed at the elastic tail of the magnetic body

(7) $$\frac{\partial^{2}}{\partial x^{2}}\left(E_{i}I_{i}\frac{\partial^{2}\varphi_{i}}{\partial x^{2}}\right)\;\;{|}_{x=0}\;\;=F_{{\mathrm{reac}}_{i}}\;\;\;\mathrm{and}\;\;\;E_{i}I_{i}\frac{\partial^{2}\varphi_{i}}{\partial x^{2}}{|}_{x=0}=T_{{\mathrm{reac}}_{i}}$$

where $F_{{\mathrm{reac}}_{i}}$ and $T_{{\mathrm{reac}}_{i}}$ denote the reaction force along *y*‐axis and torque along *z*‐axis of the *i*th tail, respectively. The reaction forces were calculated based on the force equilibrium at the elastic joints between head and tails. The net force due to the elasto‐hydrodynamics of the elastic tails and the net force due to the magneto‐hydrodynamics of the rigid head balanced each other at the elastic joints. Thus, it yielded the boundary conditions represented in Equation (7). The net local hydrodynamic force acting on the flexible tail with any arbitrary plane wave propagation was calculated using

(8) $${\left[f_{x}\;\;\;f_{y}\;\;\;f_{z}\right]}^{T}=\;\;\left[R_{i}C_{i}R_{i}^{T}\;\;\;-\;\;\;R_{i}C_{i}R_{i}^{T}S_{t_{i}}\right]\;\;\left[\begin{array}{c} V\\ \Omega \end{array}\right]$$

where $S_{t_{i}}$ is the skew‐symmetric matrix satisfying the cross‐products, $C_{i}$ and $R_{i}$ are the diagonal matrix of the local resistive‐force coefficients, and the rotation matrix from local Frenet–Serret coordinate frames to the inertial frame of reference of the microrobot, along the elastic tail respectively. Although formulation of the matrices were the same, different length and deformation profiles resulted in two separate set of resistance matrices for each tail. In addition, $Z_{r}$ is the resistance matrix of the microrobot given as the summation of resistance of the head, $Z_{h}$, first tail, $Z_{t_{1}}$, and the second tail $Z_{t_{2}}$, $Z_{r}\;\;=\;\;Z_{h}\;\;+\;\;Z_{t_{1}}\;\;+\;\;Z_{t_{2}}$. The resistance matric of the head is given by

(9) $$Z_{h}=\left(\begin{array}{cc} D_{\mathrm{tran}} & -D_{\mathrm{tran}}S_{b}\\ S_{b}D_{\mathrm{tran}} & D_{\mathrm{rot}} \end{array}\right)$$

where $D_{\mathrm{tran}}$ and $D_{\mathrm{rot}}$ are diagonal matrices of translational and rotational resistive‐force coefficients of the body, and $S_{b}$ is the skew symmetric‐matrix signifying the crossproducts. The resistance matrices of the tails are given by

(10) $$Z_{t_{i}}=\int_{0}^{l_{t_{i}}}\left(\begin{array}{c} R_{i}C_{i}R_{i}^{T}-R_{i}C_{i}R_{i}^{T}S_{t_{i}}\\ S_{t_{i}}R_{i}C_{i}R_{i}^{T}-S_{t_{i}}R_{i}C_{i}R_{i}^{T}S_{t_{i}} \end{array}\right)\;dl_{t_{i}}\;\;\mathrm{for}\;\;i=1,2$$

where $l_{t_{i}}$ is the length of the *i*th tail.

*Magnetic Actuation*: An orthogonal configuration of four electromagnetic coils was used to exert periodic magnetic torque on the soft microrobots. Each electromagnetic coil (inner diameter 20 mm, outer diameter 40 mm, and length 80 mm) had 3200 turns and thickness of the wire was 0.7 mm, and generated maximum magnetic field of 18 mT in the workspace using current input of 1 A (*I*_max_). The input currents on the coils are modeled as

(11) $$I_{\mathrm{cA}}\;\;=\;\;I_{\mathrm{cC}}\;\;=\;\;I_{\max}\sin\left(\Omega_{z}\;\;+\;\;\frac{\pi }{4}\cos\;(2\pi \omega t)\right)$$

where *I*_cA_ and *I*_cC_ are the current inputs to two collinear and opposite electromagnetic coils A and C, respectively. Further, ω and Ω_*z*_ are frequency and the *z*‐component of the rigid body rotation, respectively. The current inputs to the other collinear and opposite electromagnetic coils B (*I*_cB_) and D (*I*_cB_) are given by

(12) $$I_{\mathrm{cB}}\;\;=\;\;I_{\mathrm{cD}}\;\;=\;\;I_{\max}\cos\left(\Omega_{z}\;\;+\;\;\frac{\pi }{4}\cos\;(2\pi \omega t)\right)$$

Equations (11) and (12) represent the inputs supplied to the electromagnetic coils and the hydrodynamic model. These currents were mapped onto components of the magnetic field (B) using

(13) $$B_{x}\;\;=\;\;{\left(\frac{5}{4}\right)}^{\frac{3}{2}}\frac{B_{0}}{4\pi }\;\;\int_{0}^{2\pi }\;\left(1\;\;-\;\;\frac{y}{r}\cos\theta -\frac{z}{r}\sin\theta \right)\;F_{0}^{-\frac{3}{2}}\;d\theta$$

where *B*_*x*_ is the magnetic field along *x*‐axis and *r* is the inner radius of the electromagnetic coil. Moreover, *B*_0_ is given by

(14) $$B_{0}\;\;=\;\;{\left(\frac{4}{5}\right)}^{\frac{3}{2}}\;\;\frac{\mu_{0}NI_{c}}{r}$$

In (14), μ_0_, *N*, and *I*_c_ are the permeability of the iron core, number of turns of each coil, and the input current to each of the electromagnetic coils, respectively. Further, *F*_0_ is given by

(15) $$F_{0}\;\;=\;\;{\left(\frac{x}{r}\right)}^{2}+{\left(\frac{y}{r}-\cos\theta \right)}^{2}+{\left(\frac{z}{r}-\sin\theta \right)}^{2}$$

The magnetic field along *y*‐axis (*B*_*y*_) is given by

(16) $$B_{y}\;\;=\;\;{\left(\frac{5}{4}\right)}^{\frac{3}{2}}\frac{B_{0}}{4\pi }\;\;\int_{0}^{2\pi }\;\left(\frac{x}{r}\frac{\cos\theta }{F_{0}^{\frac{3}{2}}}\right)\;\;d\theta$$

Finally, the magnetic field along *z*‐axis (*B*_*z*_) is given by

(17) $$B_{z}\;\;=\;\;{\left(\frac{5}{4}\right)}^{\frac{3}{2}}\frac{B_{0}}{4\pi }\;\int_{0}^{2\pi }\;\left(\frac{x}{r}\frac{\sin\theta }{F_{0}^{\frac{3}{2}}}\right)\;\;d\theta$$

## Conflict of Interest

The authors declare no conflict of interest.

## Supporting information

Supplementary

Supplementary

Supplementary
